# β-lactam infusion strategies in critical illness: is continuous superior to extended infusion? A systematic review and network meta-analysis

**DOI:** 10.3389/fepid.2026.1883520

**Published:** 2026-09-18

**Authors:** Fei Peng, Ziyi Tang, Zihan Hu, Xing Zhou, Liqin Kang, Wei Chang, Qin Sun, Jianfeng Xie, Haibo Qiu, Yi Yang

**Affiliations:** 1 Jiangsu Provincial Key Laboratory of Critical Care Medicine, Department of Critical Care Medicine, Zhongda Hospital, School of Medicine, Southeast University, Nanjing, China; 2 School of Life Sciences and Chemical Engineering, Jiangsu Second Normal University, Nanjing, China

**Keywords:** continuous infusion, extended infusion, intermittent infusion, network meta-analysis, β-lactam antibiotics

## Abstract

**Background:**

Prolonged β-lactam infusion is common in critical care, but whether continuous infusion (CI) improves outcomes more than extended infusion (EI) is unclear.

**Methods:**

We conducted a systematic review and random-effects Bayesian network meta-analysis of randomized controlled trials in critically ill adults comparing intravenous β-lactam administration by continuous infusion (24-hour infusion), extended infusion (≥3 h per dose), and intermittent infusion (≤1 h per dose). We searched PubMed, EMBASE, Web of Science, and the Cochrane Library from January 1, 2000, to July 25, 2025. Two reviewers independently screened records, extracted data, and assessed risk of bias. Relative risks (RRs) and mean differences (MDs) were synthesized using random-effects models. Network meta-analysis used Markov chain Monte Carlo methods in R, with complementary pairwise analyses in Stata. Certainty of evidence was assessed using GRADE for network meta-analysis.

**Results:**

Twenty-five randomized trials (*N* = 9,915) were included; no direct CI vs. EI trials were identified. Versus II, CI reduced mortality (RR 0.85, 95% CI 0.66–0.98; moderate certainty); EI had uncertain mortality effects (RR 0.80, 95% CI 0.60–1.10; low certainty). Clinical cure improved versus II for CI (RR 1.10, 95% CI 1.03–1.20) and EI (RR 1.28, 95% CI 1.07–1.55). ICU length of stay was uncertain (CI vs. II: MD −0.47 days, 95% CI −2.10–1.28; EI vs. II: MD −1.32 days, 95% CI −4.65–2.52). Indirect CI vs. EI comparisons showed no significant differences in mortality (RR 1.06, 95% CI 0.73–1.44), clinical cure (RR 0.86, 95% CI 0.70–1.05), or ICU length of stay (MD 0.86 days, 95% CI −3.32–4.54). Bayesian posterior probability analysis suggested a 93.2% probability that EI is superior to CI for clinical cure.

**Conclusions:**

CI did not demonstrate greater clinical benefit than EI. CI can reduce patient mortality, and both CI and EI appear to improve clinical cure rates compared with II. CI conferred no additional benefit over EI, suggesting that EI may be sufficient as a prolonged-infusion strategy in clinical practice. However, this finding is based on indirect evidence. The apparent advantage of EI should therefore be regarded as hypothesis-generating and requires confirmation in adequately powered head-to-head randomized controlled trials.

**Systematic Review Registration:**

https://www.crd.york.ac.uk/PROSPERO/view/CRD42023452814, PROSPERO CRD42023452814.

## 1 Introduction

Sepsis remains one of the most common critical illnesses in intensive care units (ICUs) and is associated with high morbidity and mortality rates (1, 2). Early and appropriate antibiotic administration is among the most important strategies for reducing morbidity and mortality (3). However, appropriate antibiotic therapy can be challenging in the ICU for several reasons. The natural course of sepsis and physiological changes during management may alter antibiotic pharmacokinetics and influence outcomes in critically ill patients.

β-lactam antibiotics are widely prescribed for sepsis in the ICU because of their broad antibacterial spectrum and relatively few adverse effects (4, 5). The clinical effectiveness of β-lactams depends on the percentage of time that free plasma concentrations remain above the causative pathogen's minimum inhibitory concentration (%fT > MIC), reflecting their time-dependent bactericidal activity (6). In critically ill patients, profound and dynamic pathophysiological changes substantially alter the volume of distribution and clearance of β-lactams, leading to highly variable exposure and an increased risk of subtherapeutic concentrations with conventional intermittent infusion (II, usually infused over 0.5–1 h) regimens (7, 8). On this mechanistic basis, continuous infusion (CI, typically given as a 24-h infusion following a loading dose) may provide more sustained and stable target attainment than extended infusion (EI, usually infused over 3–4 h, corresponding to ∼40%–50% of the dosing interval), potentially improving infection control in critically ill patients.

However, from clinical and organizational perspectives, EI has been adopted more widely than CI in many ICUs. CI is operationally more challenging because it usually requires a dedicated intravenous line to avoid incompatibility with other continuously infused drugs (9), while the limited stability of many β-lactams in infusion bags over 24 h (particularly meropenem) necessitates more frequent replacement of antibiotic solutions (10). These factors increase nursing workload, catheter utilization, and consumption of infusion devices and disposables, thereby raising overall costs and patients’ financial burden. In contrast, EI is typically administered through existing central or peripheral venous access, allowing conventional dosing intervals to be maintained while extending each infusion, thereby reducing the need for dedicated intravenous lines and the frequency of infusion pump and administration-set changes.

Direct evidence comparing CI and EI remains limited, and current guidelines do not clearly distinguish between these strategies (11). Previous meta-analyses have often combined CI and EI as prolonged infusion (PI) (12–14), based on the principle that PI increases time above the MIC, thereby enhancing β-lactam activity (11). Although this approach suggests that PI can improve outcomes overall (12), comparing CI and EI separately remains important because their concentration–time profiles differ. Therefore, we conducted this network meta-analysis to compare CI, EI, and II pairwise. These comparisons are important for optimizing antibiotic therapy in critically ill patients and providing more precise clinical guidance.

## 2 Methods

### 2.1 Search strategy and selection criteria

We conducted a systematic review and network meta-analysis to compare three intravenous β-lactam infusion strategies in critically ill patients: continuous infusion (CI), extended infusion (EI), and intermittent infusion (II). Two reviewers (FP and ZT) independently searched studies published in English in PubMed, Web of Science, EMBASE and the Cochrane Library, between 1 Jan, 2000 and 25 July, 2025. Search terms are detailed in the Supplementary Table 1. We did not search abstracts presented in the international conferences. The reference lists of the articles and relevant reviews retrieved were reviewed in search of additional studies using the EndNote X9.

Any randomized controlled trials (RCTs) enrolling critically ill adults in an ICU that evaluated the comparative effectiveness and safety of β-lactam antibiotics administered as EI (lasting ≥3 h per dose) or CI (24 h of uninterrupted infusion, typically following a loading dose) versus II (bolus or ≤1 h per dose) was eligible for inclusion (15). Trials directly comparing CI with EI were also eligible. Studies were excluded if they were not RCTs, reported only pharmacokinetic/pharmacodynamic outcomes, or were protocol papers. This meta-analysis was registered in PROSPERO (CRD42023452814) and is reported in accordance with the PRISMA guidelines for network meta-analysis (16, 17).

### 2.2 Data extraction and quality assessment

Two investigators (FP, ZT, ZH and LK) independently read and assessed quality of the studies. Data extracted included authors, years of publication, study design, antibiotics, administration strategies of intravenous, number and type of participants, outcome measures, and study results. Binary outcomes from each study were extracted as counts, whereas continuous outcomes were extracted as means and standard deviations with variance estimated from interquartile ranges, or ranges (18).

Risk of bias was assessed using the Cochrane Risk of Bias Assessment Tool. The risk of publication bias for each outcome was assessed by visual inspection of funnel plots and Egger's test for funnel plot asymmetry (19). If asymmetry was detected, a trim and fill analysis was conducted. In doubtful cases the opinion of a third researcher (WC, QS and JX) was requested to reach consensus.

### 2.3 Outcomes

The primary outcome was all-cause mortality at any timepoint, defined according to each study's methods. If ICU mortality were available, this was included in the analysis. If not, any other mortality data was included. Secondary outcomes were clinical cure, length of ICU stay and length of hospital stay. Clinical cure was extracted according to the definition used in each study, and the corresponding definitions and assessment times are summarized in Supplementary Table 3.

### 2.4 Statistical analysis

#### 2.4.1 Data synthesis

For binary outcomes, we extracted the number of events and the total number of patients in both the intervention and control groups. The relative risk (RR) was used as the effect measure, with 95% confidence intervals provided. For continuous variables, we extracted the mean and standard deviation (SD) reported in the articles and used the mean difference (MD) as the effect measure, also with 95% confidence intervals. If data were reported as medians with ranges or interquartile ranges, we converted them to means and SDs according to established methods (18).

We performed a Bayesian network meta-analysis using R (version 4.4.3), integrating clinical trial data via Markov Chain Monte Carlo methods. The packages gemtc and rjags were used for the analysis. We used gemtc's default vague priors: relative treatment effects on the log-RR scale were assigned zero-centered normal priors (SD = 15 × om.scale), and the between-study heterogeneity SD was assigned a Uniform (0, om.scale) prior, with om.scale automatically determined from the outcome data. Pairwise meta-analyses were conducted using Stata 18.0 (StataCorp LP, College Station, USA). Given the observed heterogeneity in study design and settings among the included studies, all analyses were performed using a random-effects model. Heterogeneity was assessed by visual inspection of forest plots and by calculating the *I*^2^ statistic (20). In addition, a trial sequential analysis (TSA) was conducted for the primary outcome to assess the risk of random errors and to calculate the required number of participants (required information size, RIS). The RIS in TSA was based on a 5% risk of type I error and a 20% risk of type II error (power of 80%). Analyses were performed using TSA (version 0.9.5.10).

#### 2.4.2 Subgroup and sensitivity analyses

When at least two trials contributed data to a subgroup, we conducted prespecified subgroup analyses and meta-regression for the primary outcome, and calculated Bayesian posterior probabilities (R version 4.4.3; gemtc package). If a subgroup effect modification was identified, we used the Instrument for assessing the Credibility of Effect Modification Analyses (ICEMAN) tool to evaluate the credibility of the observed effect modification (21). The predefined subgroups included population (sepsis or septic shock vs. pneumonia), antibiotic class (carbapenems, piperacillin–tazobactam, cephalosporins, penicillins, or other), study setting (single-center vs. multicenter), national economic status (classified as high-income vs. low- and middle-income regions according to World Bank criteria, https://datahelpdesk.worldbank.org), reported Gram stain of pathogens (Gram-negative vs. positive), masking status (blinded vs. open-label), time point of mortality recording (ICU mortality vs. other short-term mortality), and study scope (clinical vs. pharmacokinetics). Given that BLING III contributed approximately 70% of the overall study population, we also performed a *post hoc* leave-one-out sensitivity analysis by excluding this trial and repeating the random-effects Bayesian network meta-analysis.

### 2.5 Certainty of evidence

We assessed the certainty of evidence for all outcomes according to the Grading of Recommendations Assessment, Development and Evaluation (GRADE) approach for network meta-analyses (22). For direct comparisons, we evaluated risk of bias, inconsistency, indirectness, publication bias, and imprecision to determine the overall certainty of evidence. For indirect comparisons, the initial certainty was based on the ratings from the contributing direct comparisons, with additional assessment of intransitivity and imprecision. The evaluation process is detailed in Supplementary Table 4.

## 3 Results

### 3.1 Study characteristics

We identified 2,516 articles through database searching, of which 191 underwent full-text review. Ultimately, 25 studies met our inclusion criteria (23–47). Two articles reported the same outcomes and were therefore combined as a single study (31, 48) (Figure 1).

**Figure 1:**
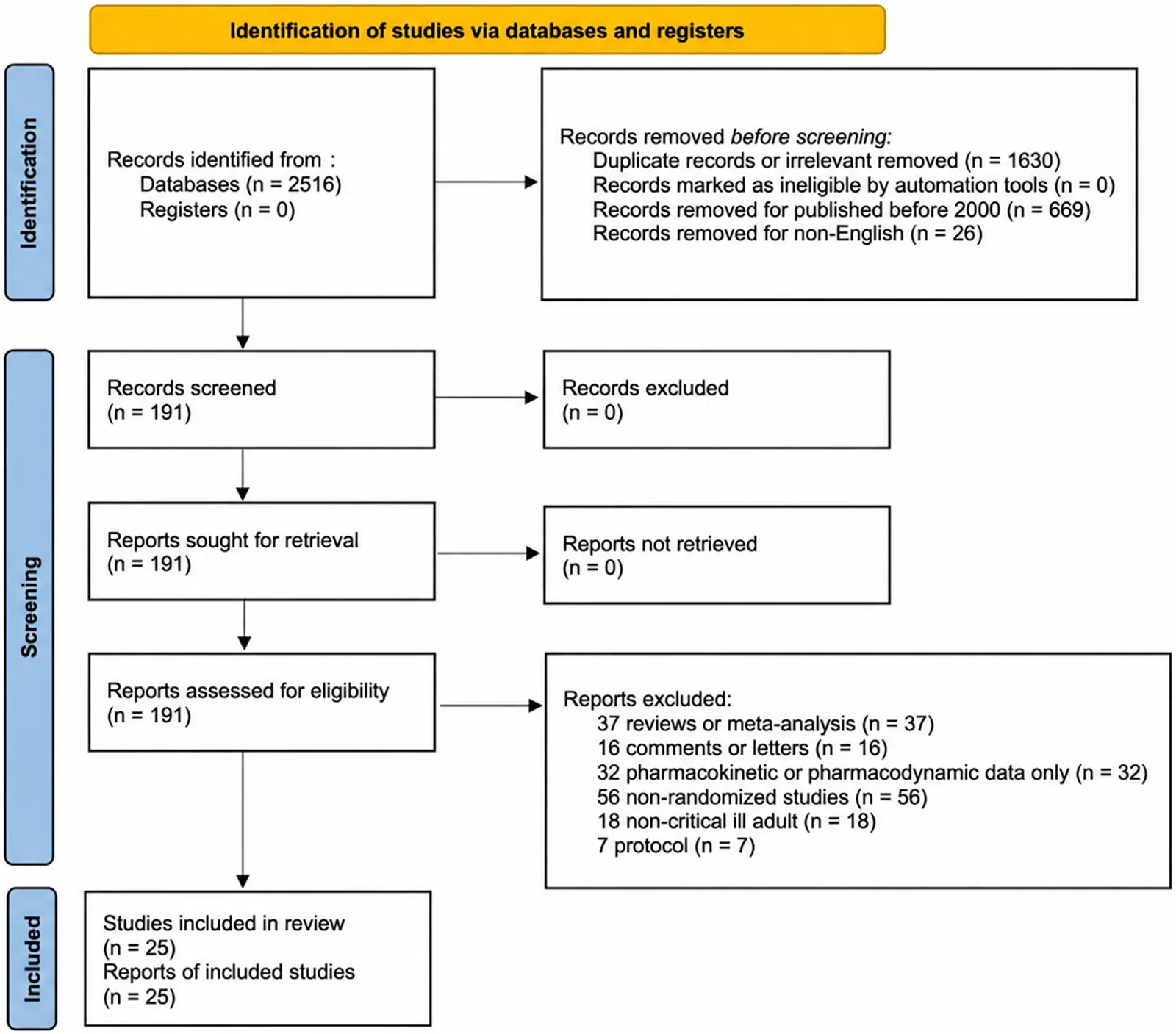
Study flow.

Detailed characteristics of the included studies and network plots are provided in Supplementary Tables 2, 3 and Supplementary Figure 1. All studies assessed clinical outcomes, encompassing a total of 9,915 patients. Eighteen studies (23, 31–47), with a combined total of 9,095 patients, compared CI with II. Seven studies (24–30), involving 825 patients, compared EI with II. No studies comparing CI with EI were identified (Table 1). The mean patient age was 59 years. The main populations studied included patients with sepsis or septic shock (*n* = 12) (29, 32, 33, 35, 36, 38–40, 43, 44, 46, 47), pneumonia (*n* = 7) (23–25, 27, 31, 34, 37), intra-abdominal infection (*n* = 2) (41, 42), Gram-negative infections (*n* = 1) (45), and neutropenia-associated fever (*n* = 1) (28). The antibiotics investigated included carbapenems (*n* = 7) (24, 25, 33, 36, 37, 40, 46), cephalosporins (*n* = 7) (27, 28, 30, 33, 35, 41, 45), piperacillin–tazobactam (*n* = 7) (23, 31, 32, 34, 35, 38, 47), penicillins (*n* = 3) (29, 39, 42), and others. The total daily dose of β-lactam antibiotics varied both within and between studies. The duration of antibiotic therapy also differed across the reported studies.

**Table 1:** Basic characteristics of the included studies.

Authors	Design	Antibiotics	Patients	CI/EI vs. II N.	Age CI/EI^a^, yr	Age II^a^, yr	Male CI/EI (*N*, %)	Male II (*N*, %)
Continuous Infusion vs. intermittent infusion								
Álvarez-Moreno et al. (32)	Randomized 1:1 multicenter double-blinded placebo-controlled study	Cefepime	sepsis	12 vs. 13	60.2 ± 16.9	54.2 ± 1.4	8 (66.7%)	4 (30.8%)
Angus et al. (47)	Randomised controlled trial	Ceftazidime	suspected septic aemic melioidosis	10 vs. 11	48 (29 ± 58)	43 (27 ± 73)	4 (36.4%)	9 (90%)
Chytra et al. (46)	Single-center, prospective, randomized, open-label comparative study	Meropenem	sepsis	106 vs. 108	44.9 ± 17.8	47.2 ± 16.3	78 (65.0%)	83 (69.2%)
Cotrina-Luque et al. (45)	Randomized, double-blind parallel-group clinical trial	Piperacillin-tazobactam	suspected or proven P.aeruginosa	40 vs. 38	64.3 ± 14.3	63.8 ± 17.3	26 (65%)	20 (52.6%)
Dulhunty et al. (44)	Prospective, multicenter, double-blind, concealed, randomized controlled trial	Piperacillin-tazobactam, meropenem, and ticarcillin-clavulanate	severe sepsis	30 vs. 30	54 ± 19	60 ± 19	23 (76.7%)	19 (63.3%)
Dulhunty et al. (43)	Prospective, multicenter, double-blind, double-dummy, randomized controlled trial (BLING II)	Piperacillin-tazobactam, ticarcillin-clavulanate or meropenem	severe sepsis	212 vs. 220	64 (54–72)	65 (53–72)	130 (61.3%)	135 (61.4%)
Dulhunty et al. (35)	Open-label, randomized clinical trial (BLING III)	Piperacillin-tazobactam or meropenem	sepsis	3474 vs. 3507	59.3 ± 16.4	59.6 ± 16.1	2308 (66%)	2300 (65%)
Georges et al. (23)	Prospective, open, randomized study	Cefepime	nosocomial pneumonopathy	26 vs. 24	50 ± 17	46 ± 24	21 (80.7%)	20 (83.3%)
Khan et al. (33)	Prospective, open-label, single-center, randomized controlled trial	Amoxicillin-clavulanate, Piperacillin-tazobactam, Imipenem-cilastatin, Meropenem	Critically ill patients with sepsis	64 vs. 58	31 (26–39)	36 (25–50)	41 (64%)	34 (58.6%)
Laterre et al. (42)	Prospective, two-centre, randomized, controlled study	Temocillin	intra-abdominal or lower respiratory tract infections caused by Enterobacteriaceae	14 vs. 14	68 ± 11	65 ± 15	11 (78.6%)	6 (42.9%)
Lau et al. (41)	Multicenter, prospective, randomized, open-label comparative study	Piperacillin-tazobactam	complicated intra-abdominal infections	128 vs. 130	50.4 ± 16.6	49.3 ± 17.8	81 (63.3%)	75 (57.7%)
Maranchic et al. (34)	prospective, randomized, open-label, randomized trial	Cefepime, Meropenem, Piperacillin–Tazobactam	Severe pneumonia caused by Gram-negative bacteria	19 vs. 16	67 (49–74)	69 (61–73)	4 (80%)	8 (62%)
McNabb et al./ Nicolau et al. (31, 48)	Prospective, open-label, randomized trial	Ceftazidime	nosocomial pneumonia	17 vs. 18	46 ± 16	56 ± 20	10 (58.8%)	13 (72.2%)
Monti et al. (40)	Double-blind, randomized clinical trial (MERCY)	Meropenem	sepsis or septic shock	303 vs. 304	65.5 ± 14	63.4 ± 15	195 (64%)	209 (69%)
Rafati et al. (39)	Prospective, randomised controlled trial	Piperacillin	Sepsis	20 vs. 20	50.1 ± 22.2	48.0 ± 20.7	12 (60%)	15 (75%)
Roberts et al. (38)	Open-label, randomized controlled pilot study	Ceftriaxone	sepsis	29 vs. 28	43 ± 19	52 ± 16	16 (55%)	17 (61%)
Sakka et al. (37)	Randomized, Controlled Trial	Imipenem-cilastatin	ICU-acquired pneumonia	10 vs. 10	62 ± 16	59 ± 16	5 (50%)	6 (60%)
Zhao et al. (36)	Single-center, prospective, randomized, comparative study	Meropenem	severe sepsis or septic shock	25 vs. 25	68.0 ± 15.4	67.0 ± 12.2	10 (40%)	11 (44%)
Extended infusion vs. intermittent infusion								
Abdul-Aziz et al. (26)	Prospective, two-centre, open labelled RCT (BLISS)	Cefepime, meropenem or piperacillin/tazobactam	severe sepsis	70 vs. 70	54 (42–63)	56 (41–68)	46 (66%)	50 (71%)
Ammar and Abdalla (25)	Prospective, randomized, nonblinded, controlled, single center trial	Meropenem	ventilator associated pneumonia	30 vs. 30	55.8 ± 3.5	55.5 ± 3.8	24 (80%)	23 (76.7%)
Bao et al. (27)	Randomized, parallel study	Piperacillin-tazobactam	hospital-acquired pneumonia	25 vs. 25	69.7 ± 5.9	67.0 ± 7.8	14 (56%)	15 (60%)
Fan et al. (28)	Single-center, open-label, prospective study	Piperacillin/tazobactam	bacterial infection or neutropenic fever	182 vs. 185	69 ± 14.6	70 ± 13.8	126 (69.2%)	121 (65.4%)
Lips et al. (24)	Single-centre, randomised, open-label, comparative study	Imipenem/cilastatin	hospital-acquired pneumonia	10 vs. 9	63 ± 21	57 ± 16	5 (55%)	9 (90%)
Mirjalili et al. (29)	Randomized assessor-blinded clinical trial	Ampicillin/sulbactam	sepsis and septic shock	68 vs. 68	53.8 ± 15.8	53.1 ± 16.2	37 (54.5)	38 (55.9%)
Naiim et al. (30)	Prospective non-blinded randomized comparative study	Piperacillin/tazobactam	gram-negative bacterial infections	26 vs. 27	57.8 ± 15.4	53.0 ± 17.6	12 (46%)	14 (52%)

ᵃAge data are presented as mean ± SD or median (interquartile range). Full study characteristics are provided in the Supplementary Material.

CI, continuous infusion; EI, extended infusion; II, intermittent infusion; SD, standard deviation.

### 3.2 Outcomes

#### 3.2.1 All-cause mortality

Except for one study (31), all included studies reported mortality data of different categories. These included 28-day mortality (*n* = 1) (40), 30-day mortality (*n* = 1) (26), 90-day mortality (*n* = 2) (33, 35), ventilator-associated mortality (*n* = 1) (25), in-hospital mortality (*n* = 3) (28, 30, 32), ICU mortality (*n* = 7) (24, 29, 36, 42–44, 46), and nine studies that did not specify the exact time point of mortality (23, 27, 34, 37–39, 41, 45, 47). Overall, CI was associated with a significantly reduced risk of all-cause mortality compared to II (RR: 0.85, 95% CI: 0.66–0.98, *I*^2^ = 23.0%). There was no statistically significant difference between EI and II (RR: 0.80, 95% CI: 0.60–1.10, *I*^2^ = 0%), nor between CI and EI (RR: 1.06, 95% CI: 0.73–1.44) (Figure 2). The results of pairwise meta-analyses remained consistent (Supplementary Figure 2). Bayesian posterior probability analysis suggested that EI had a 63.7% probability of being superior to CI in reducing all-cause mortality (Supplementary Figure 10).

**Figure 2:**
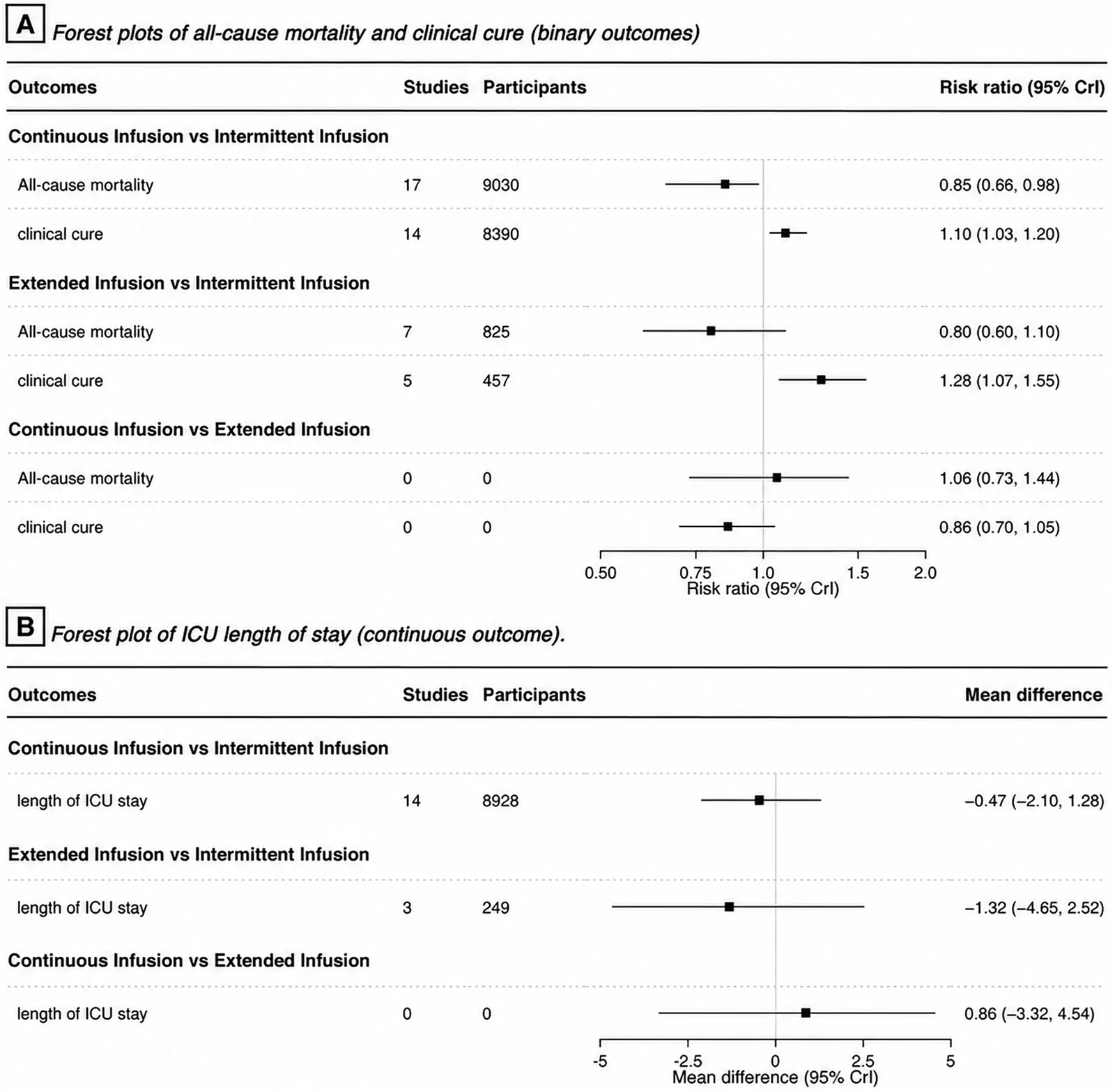
Forest plots of binary **(A)** and continuous **(B)** outcomes. CI, continuous infusion; EI, extended infusion; II, intermittent infusion; CrI, credible interval.

#### 3.2.2 Clinical cure

The definitions and assessment times of clinical cure varied across studies, ranging from complete resolution of infection-related signs and symptoms to broader measures of clinical improvement or treatment success. A total of 19 studies reported clinical cure outcomes (23, 25–27, 29–36, 38, 41–46), including 8,847 patients. Among them, 8,390 patients were in the CI versus II group, and 457 patients were in the EI versus II group. CI was associated with a significantly higher clinical cure rate compared to II (RR: 1.10, 95% CI: 1.03–1.20, *I*^2^ = 0%). EI also improved clinical cure rates compared to II (RR: 1.28, 95% CI: 1.07–1.55, *I*^2^ = 0%). However, there was no statistically significant difference between CI and EI (RR: 0.86, 95% CI: 0.70–1.05) (Figure 2). In pairwise meta-analysis, there was no difference observed between CI and II (RR: 1.20, 95% CI: 0.97–1.48, *I*^2^ = 0%) (Supplementary Figure 3). Bayesian posterior probability analysis indicated a 93.2% probability that EI achieves a higher clinical cure rate than CI (Figure 3).

**Figure 3:**
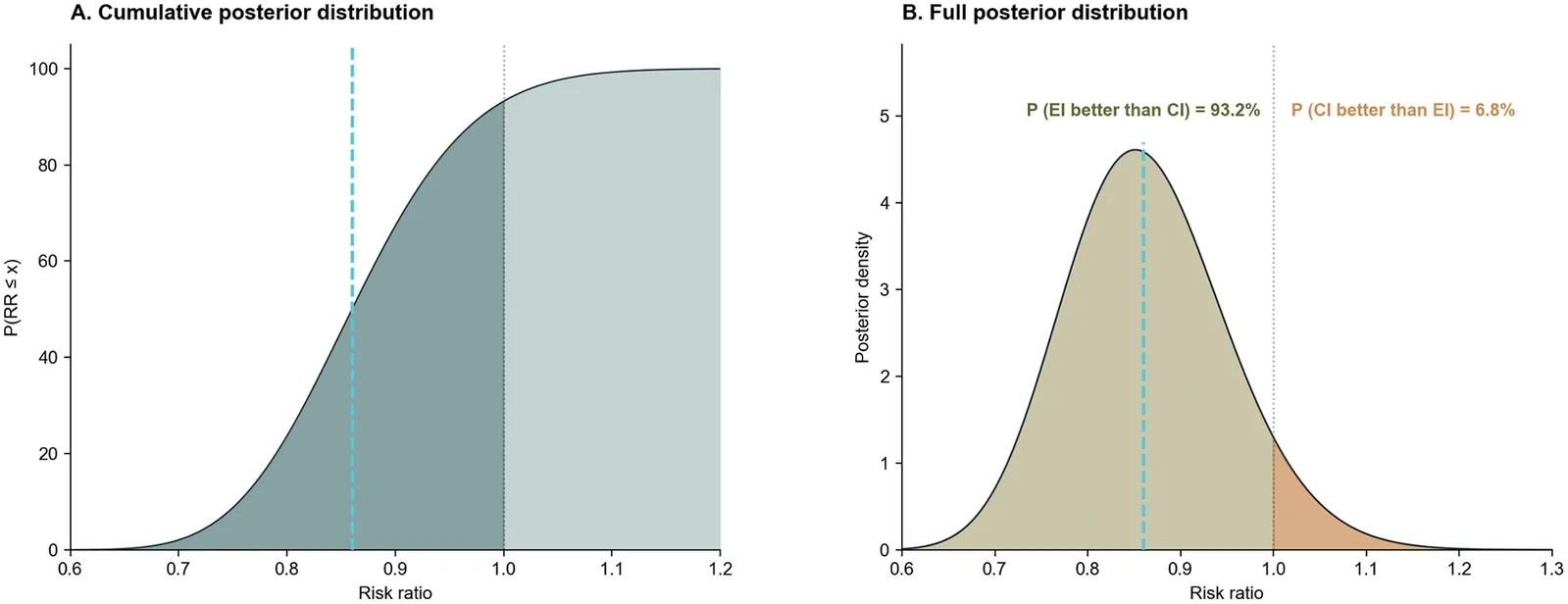
Bayesian posterior distributions for the indirect comparison of continuous infusion versus extended infusion of β-lactam antibiotics on clinical cure. **(A)** The cumulative posterior distribution of the estimated RR, with the *y*-axis corresponding to the probability that the RR is less than or equal to the value on the *x*-axis. The darker blue-gray area indicates the cumulative probability up to RR = 1. The dashed vertical line indicates the median. **(B)** The full posterior distribution of the estimated RR, with the dashed vertical line indicating the median value. The tan area (RR ≥ 1) represents the posterior probability that extended infusion achieves a higher clinical cure rate than continuous infusion (extended infusion is superior), and the orange area (RR < 1) represents the probability that continuous infusion achieves a higher clinical cure rate (continuous infusion is superior). The dotted line at an RR of 1 indicates no treatment effect. The posterior probability that extended infusion achieves a higher clinical cure rate than continuous infusion is 93.2%.

#### 3.2.3 Length of ICU/hospital stay

Among the included studies, 17 reported ICU length of stay outcomes (23, 25, 29–34, 36–38, 40, 41, 43, 44, 46). There was no effect on ICU length of stay for any of the comparisons: CI versus II (MD: −0.47 day, 95% CI: −2.10–1.28, *I*^2^ = 87.0%), EI versus II (MD: −1.32 day, 95% CI: −4.65–2.52, *I*^2^ = 74.3%), or CI versus EI (MD: 0.86 day, 95% CI: −3.32–4.54) (Figure 2; Supplementary Figure 4). A total of seven studies reported hospital length of stay (34, 38, 40, 41, 43, 44, 46). The pairwise meta-analysis found no significant difference in hospital length of stay between CI and II (MD: 0.09 day, 95% CI: −0.03–0.22, *I*^2^ = 61.4%) (Supplementary Figure 5).

#### 3.2.4 Subgroup and sensitivity analyses

We performed subgroup analyses for the CI vs. II, EI vs. II (Supplementary Figure 6), and CI vs. EI (Figure 4) comparisons. Because subsequent meta-regression did not identify any statistically significant subgroup modification effects (*p* > 0.05), the ICEMAN tool was not applied. The Bayesian posterior distributions of the interaction terms are presented in Supplementary Table 5. Notably, no subgroup differences were observed in the subgroup analyses of CI vs. II (Figure 4).

**Figure 4:**
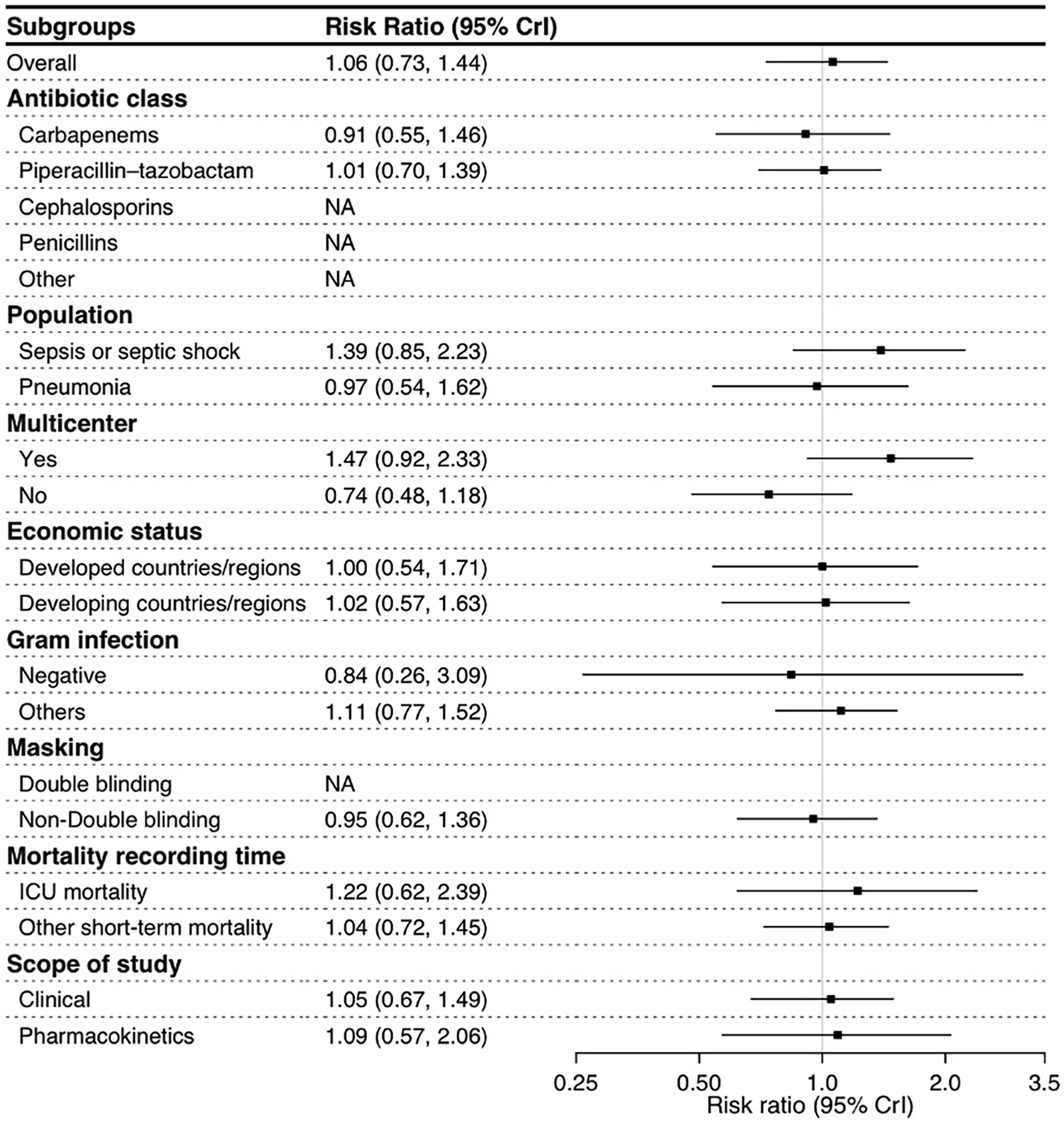
Subgroup analyses for all-cause mortality comparing continuous versus extended β-lactam infusion. CrI, credible interval; NA, not available.

Given the substantial contribution of BLING III, we conducted a *post hoc* sensitivity analysis excluding this trial. After exclusion, CI remained associated with lower mortality (RR 0.78, 95% CrI 0.60–0.96) and higher clinical cure (RR 1.09, 95% CrI 1.01–1.23) compared with II. The indirect CI-versus-EI estimates remained nonsignificant for mortality and clinical cure (Supplementary Table 6).

#### 3.2.5 Trial sequential analysis

A *post hoc* TSA for mortality including all eligible trials showed that, for both the CI vs. II and EI vs. II comparisons, the cumulative Z-curves reached the required information size (RIS). For CI vs. II, the cumulative Z-curve crossed the statistical significance boundary, whereas for EI vs. II, the final Z-value remained below the conventional significance threshold and did not cross the trial sequential monitoring boundary, indicating that the mortality effect of EI versus II remained uncertain (Supplementary Figure 7).

#### 3.2.6 Grading of recommendations assessment, development and evaluation

Our GRADE assessment showed that, compared with II, CI with β-lactam antibiotics had moderate certainty for reducing mortality and improving clinical cure rates among critically ill patients. The certainty of evidence for the effect of EI on mortality was low, but EI had moderate certainty for improving clinical cure rates. For ICU length of stay, both CI and EI demonstrated low certainty of evidence (Table 2).

**Table 2:** Grading of recommendations assessment, development and evaluation (GRADE) summary of findings.

Outcomes	No. of trials/no. of participants	Relative effect (95%CI)	Absolute effect estimates		Risk difference	(Certainty of evidence)^a^
			Control	Intervention		
All-cause mortality						
Continuous infusion vs. intermittent infusion	17/9030	RR: 0.85 (0.66 to 0.98)	257/1000 patients	218/1000 (170 to 252)	39 fewer per 1000 (87 to 5 fewer)	Moderate
Extended infusion vs. intermittent infusion	7/825	RR: 0.80 (0.60 to 1.10)	300/1000 patients	240/1000 (180 to 330)	60 fewer per 1000 (120 fewer to 30 more)	Low
Continuous infusion vs. extended infusion	0/0	RR: 1.06 (0.73 to 1.44)	not estimated			Very Low
Clinical cure						
Continuous infusion vs. intermittent infusion	14/8390	RR: 1.10 (1.03 to 1.20)	521/1000 patients	573/1000 (537 to 625)	52 more per 1000 (16 to 104 more)	Moderate
Extended infusion vs. intermittent infusion	5/457	RR: 1.28 (1.07 to 1.55)	441/1000 patients	564/1000 (472 to 684)	123 more per 1000 (31 to 243 more)	Moderate
Continuous infusion vs. extended infusion	0/0	RR: 0.86 (0.70 to 1.05)	not estimated			Very Low
Length of ICU stay						
Continuous infusion vs. intermittent infusion	14/8928	MD: −0.47 (−2.10 to 1.28)	13.59 days	13.12 (11.49 to 14.87)	0.47 lower (2.1 lower to 1.28 higher)	Very Low
Extended infusion vs. intermittent infusion	3/249	MD: −1.32 (−4.65 to 2.52)	16.39 days	15.07 (11.74 to 18.91)	1.32 lower (4.65 lower to 2.52 higher)	Low
Continuous infusion vs. extended infusion	0/0	MD: 0.86 (−3.32 to 4.54)	not estimated			Very Low

ᵃAccording to the GRADE approach, certainty of evidence is classified into four levels: high, moderate, low, and very low. Specific reasons for downgrading are provided in the Supplementary Material.

RR, risk ratio; MD, mean difference; CI, confidence interval; ICU, intensive care unit.

#### 3.2.7 Risk of bias

Most studies exhibited a high risk of bias, with performance bias being the primary source (Supplementary Figure 8). The funnel plot did not show any obvious publication bias, as studies were relatively evenly distributed on both sides of the line of unity (Supplementary Figure 9).

## 4 Discussion

Although PI of β-lactam antibiotics is recommended for sepsis, its clinical effectiveness remains controversial. In our systematic review and network meta-analysis of 25 studies, CI significantly reduced mortality and improved clinical cure, while EI also improved clinical cure; no significant difference was observed between CI and EI. Because of the low certainty of evidence, the effects of CI or EI on ICU or hospital length of stay remain unclear. Our findings have important implications for clinical practice, as they provide clear guidance on the two strategies of PI of β-lactam antibiotics. Although BLING III contributed substantially to the precision of the estimates, the direction and overall interpretation of the mortality and clinical-cure findings were not materially altered after its exclusion. Nevertheless, the large contribution of this single trial should be considered when interpreting the results.

β-Lactam antibiotics are time-dependent bactericidal agents whose efficacy depends on the duration for which free drug concentrations remain above the pathogen's MIC (%fT > MIC) (49). Conventional II achieves high peak levels but allows concentrations to fall below the MIC for considerable periods, particularly in critically ill patients, thereby risking underexposure. In contrast, EI (2–4 h) and CI (up to 24 h) prolong time above the MIC, improving bacterial killing and suppressing regrowth, which may underlie their observed advantages over II (50).

Our study further showed no significant difference between CI and EI in key outcomes. This may be because both strategies share the primary goal of prolonging β-lactam exposure and increasing fT > MIC (51). When dose, frequency, and infusion duration are sufficient to maintain drug concentrations above the target threshold for most of the dosing interval, additional clinical differences may be difficult to detect, especially with endpoints such as mortality or clinical cure. Moreover, substantial pharmacokinetic heterogeneity among critically ill patients may produce interindividual variations in drug exposure greater than differences between EI and CI. Many trials did not use systematic therapeutic drug monitoring (TDM) of β-lactams, leaving actual drug concentrations unknown and potentially obscuring true differences between the two strategies (52). To further explore the relative efficacy of CI and EI, we performed Bayesian posterior probability analyses for the indirect comparison. The Bayesian analysis indicated that EI had a 93.2% posterior probability of achieving higher clinical cure rates and a 63.7% probability of reducing mortality compared with CI. The stronger signal for clinical cure may reflect more reliable drug exposure with EI, particularly for β-lactams such as carbapenems that have limited stability during 24-hour infusion (53). However, the modest probability for mortality suggests that current evidence is insufficient to confirm a survival difference between the two strategies, and these findings should be considered hypothesis-generating.

In clinical practice, EI is often preferred because many β-lactams (especially carbapenems such as meropenem and imipenem) have limited stability during prolonged room-temperature infusion, complicating effective 24-hour CI (50). For example, meropenem is relatively unstable at room temperature, increasing the risk of degradation during CI; 3–4-hour EI regimens are therefore commonly used as a practical alternative (10). In addition, because each EI dose is infused over only a few hours, the line can be used between doses for other intravenous drugs or flushing, reducing the risk of incompatibilities (54). By contrast, CI requires round-the-clock infusion pumps and close monitoring, while patients remain continuously connected to infusion devices, increasing nursing workload and financial costs. Given that EI appears to achieve clinical outcomes comparable to CI, routine CI may offer little additional benefit over EI.

Our meta-analysis has several limitations. First, participants, healthcare providers, and investigators were aware of treatment allocation in most included RCTs, potentially introducing performance bias. Accordingly, we downgraded the certainty of evidence. Second, outcome definitions and assessment times varied across studies. Mortality was reported at different timepoints, while the definitions and assessment times of clinical cure were not identical. Pooling these outcomes may introduce clinical heterogeneity. The available data were insufficient to determine whether the mortality RR changed over time, and although subgroup analysis by mortality recording time did not identify significant effect modification, its statistical power was limited. Therefore, the pooled mortality and clinical cure findings should be interpreted with caution. Third, factors such as renal replacement therapy, albumin levels, and interindividual variability may alter free plasma antibiotic concentrations and pharmacodynamic target attainment. Finally, definitions and guidelines have evolved over time, and most RCTs used sepsis definitions and severity criteria that differ from current standards.

## 5 Conclusion

In critically ill adult patients treated with β-lactam antibiotics, continuous infusion, compared with intermittent infusion, probably reduces mortality and improves clinical cure rates, and extended infusion may also improve clinical cure rates. Our findings suggest that extended infusion may offer advantages over continuous infusion in terms of clinical cure and practical implementation. Given their comparable efficacy and the practical advantage that extended infusion can be administered through existing venous access without the need for additional infusion pumps or dedicated lines, thereby reducing nursing workload and patients’ economic burden, extended infusion may be a more clinically practical option. However, this inference is based on indirect evidence in the absence of head-to-head trials and should be confirmed in adequately powered randomized controlled trials directly comparing the two strategies.

## Data Availability

The original contributions presented in the study are included in the article/Supplementary Material, further inquiries can be directed to the corresponding author.
