# Supplementary material for: β-lactam infusion strategies in critical illness: is continuous superior to extended infusion? A systematic review and network meta-analysis
Source: Front Epidemiol. 2026 Sep 18;6:1883520. doi: 10.3389/fepid.2026.1883520 (PMC13630778; doi:10.3389/fepid.2026.1883520)
Supplement: Supplementary file 1 [file Datasheet1.docx]

β-Lactam Infusion Strategies in Critical Illness: Is Continuous Superior to Extended Infusion?

A Systematic Review and Network Meta-analysis

Supplementary Material

Supplementary Appendix. PRISMA NMA Checklist of Items to Include When Reporting A Systematic Review Involving a Network Meta-analysis

Supplementary Table 1. Search strategy

Supplementary Table 2. Characteristics of included studies

Supplementary Table 3. Study design and patient characteristics of included studies

Supplementary Table 4. Grading of recommendation assessment, development and evaluation summary of findings for all outcomes

Supplementary Table 5. Subgroup Interaction Effects on All-Cause Mortality in β-Lactam Infusion Strategies

Supplementary Table 6. Leave-one-out sensitivity analysis excluding the BLING III trial

Supplementary Figure 1. Network Plot

Supplementary Figure 2. Forest Plot of Pairwise Comparisons for All-Cause Mortality

Supplementary Figure 3. Forest Plot of Pairwise Comparisons for Clinical Cure

Supplementary Figure 4. Forest Plot of Pairwise Comparisons for Length of ICU stay

Supplementary Figure 5. Forest Plot of Pairwise Meta-Analysis for Hospital Length of Stay (CI vs II)

Supplementary Figure 6. Forest Plot of Subgroup Analyses for Primary Clinical Outcome

Supplementary Figure 7. Trial sequential analysis (TSA) of all-cause mortality comparing β-lactam infusion strategies. Continuous infusion versus intermittent infusion (A) and extended infusion versus intermittent infusion (B).

Supplementary Figure 8. Risk of Bias Assessment

Supplementary Figure 9. Funnel Plot

Supplementary Figure 10. Bayesian posterior distributions for the indirect comparison of continuous infusion versus extended infusion of β-lactam antibiotics on all-cause mortality

PRISMA NMA Checklist

**PRISMA NMA Checklist of Items to Include When Reporting A Systematic Review Involving a Network Meta-analysis**

| **Section/Topic** | **Item #** | **Checklist Item** | **Reported in Section** |
| --- | --- | --- | --- |
| **TITLE** |  |  |  |
| Title | 1 | Identify the report as a systematic review *incorporating a network meta-analysis (or related form of meta-analysis).* | Title |
|  |  |  |  |
| **ABSTRACT** |  |  |  |
| Structured summary | 2 | Provide a structured summary including, as applicable:  **Background:** main objectives  **Methods:** data sources; study eligibility criteria, participants, and interventions; study appraisal; and *synthesis methods, such as network meta-analysis.*  **Results:** number of studies and participants identified; summary estimates with corresponding confidence/credible intervals; *treatment rankings may also be discussed. Authors may choose to summarize pairwise comparisons against a chosen treatment included in their analyses for brevity.*  **Discussion/Conclusions:** limitations; conclusions and implications of findings.  **Other:** primary source of funding; systematic review registration number with registry name. | Abstract |
|  |  |  |  |
| **INTRODUCTION** |  |  |  |
| Rationale | 3 | Describe the rationale for the review in the context of what is already known*, including mention of why a network meta-analysis has been conducted.* | Introduction |
| Objectives | 4 | Provide an explicit statement of questions being addressed, with reference to participants, interventions, comparisons, outcomes, and study design (PICOS). | Introduction |
|  |  |  |  |
| **METHODS** |  |  |  |
| Protocol and registration | 5 | Indicate whether a review protocol exists and if and where it can be accessed (e.g., Web address); and, if available, provide registration information, including registration number. | Search strategy and selection criteria |
| Eligibility criteria | 6 | Specify study characteristics (e.g., PICOS, length of follow-up) and report characteristics (e.g., years considered, language, publication status) used as criteria for eligibility, giving rationale. *Clearly describe eligible treatments included in the treatment network, and note whether any have been clustered or merged into the same node (with justification).* | Search strategy and selection criteria |
| Information sources | 7 | Describe all information sources (e.g., databases with dates of coverage, contact with study authors to identify additional studies) in the search and date last searched. | Search strategy and selection criteria |
| Search | 8 | Present full electronic search strategy for at least one database, including any limits used, such that it could be repeated. | Search strategy and selection criteria; Supplementary Table 1 |
| Study selection | 9 | State the process for selecting studies (i.e., screening, eligibility, included in systematic review, and, if applicable, included in the meta-analysis). | Search strategy and selection criteria |
| Data collection process | 10 | Describe method of data extraction from reports (e.g., piloted forms, independently, in duplicate) and any processes for obtaining and confirming data from investigators. | Data extraction and quality assessment |
| Data items | 11 | List and define all variables for which data were sought (e.g., PICOS, funding sources) and any assumptions and simplifications made. | Data extraction and quality assessment |
| Geometry of the network | S1 | Describe methods used to explore the geometry of the treatment network under study and potential biases related to it. This should include how the evidence base has been graphically summarized for presentation, and what characteristics were compiled and used to describe the evidence base to readers. | Data synthesis |
| Risk of bias within individual studies | 12 | Describe methods used for assessing risk of bias of individual studies (including specification of whether this was done at the study or outcome level), and how this information is to be used in any data synthesis. | Data extraction and quality assessment |
| Summary measures | 13 | State the principal summary measures (e.g., risk ratio, difference in means). *Also describe the use of additional summary measures assessed, such as treatment rankings and surface under the cumulative ranking curve (SUCRA) values, as well as modified approaches used to present summary findings from meta-analyses.* | Data synthesis |
| Planned methods of analysis | 14 | Describe the methods of handling data and combining results of studies for each network meta-analysis. This should include, but not be limited to:   - *Handling of multi-arm trials;* - *Selection of variance structure;* - *Selection of prior distributions in Bayesian analyses; and* - *Assessment of model fit.* | Data synthesis |
| Risk of bias across studies | 15 | Specify any assessment of risk of bias that may affect the cumulative evidence (e.g., publication bias, selective reporting within studies). | Data extraction and quality assessment |
| Additional analyses | 16 | Describe methods of additional analyses if done, indicating which were pre-specified. This may include, but not be limited to, the following:   - Sensitivity or subgroup analyses; - Meta-regression analyses; - *Alternative formulations of the treatment network; and* - *Use of alternative prior distributions for Bayesian analyses (if applicable).* | Subgroup and sensitivity analyses |
|  |  |  |  |
| **RESULTS†** |  |  |  |
| Study selection | 17 | Give numbers of studies screened, assessed for eligibility, and included in the review, with reasons for exclusions at each stage, ideally with a flow diagram. | Study characteristics |
| **Presentation of network structure** | **S3** | Provide a network graph of the included studies to enable visualization of the geometry of the treatment network. | Study characteristics; Supplementary Figure 1 |
| **Summary of network geometry** | **S4** | Provide a brief overview of characteristics of the treatment network. This may include commentary on the abundance of trials and randomized patients for the different interventions and pairwise comparisons in the network, gaps of evidence in the treatment network, and potential biases reflected by the network structure. | Study characteristics; Supplementary Figure 1 |
| Study characteristics | 18 | For each study, present characteristics for which data were extracted (e.g., study size, PICOS, follow-up period) and provide the citations. | Study characteristics; Supplementary Tables 2-3 |
| Risk of bias within studies | 19 | Present data on risk of bias of each study and, if available, any outcome level assessment. | Risk of bias; Supplementary Figure 8 |
| Results of individual studies | 20 | For all outcomes considered (benefits or harms), present, for each study: 1) simple summary data for each intervention group, and 2) effect estimates and confidence intervals. *Modified approaches may be needed to deal with information from larger networks.* | Outcomes; Supplementary Tables 2-3 and Figures 2-5 |
| Synthesis of results | 21 | Present results of each meta-analysis done, including confidence/credible intervals. *In larger networks, authors may focus on comparisons versus a particular comparator (e.g. placebo or standard care), with full findings presented in an appendix. League tables and forest plots may be considered to summarize pairwise comparisons.* If additional summary measures were explored (such as treatment rankings), these should also be presented. | All-cause mortality; Clinical cure; Length of ICU/hospital stay |
| **Exploration for inconsistency** | **S5** | Describe results from investigations of inconsistency. This may include such information as measures of model fit to compare consistency and inconsistency models, *P* values from statistical tests, or summary of inconsistency estimates from different parts of the treatment network. | Grading of Recommendations Assessment, Development and Evaluation; Supplementary Table 4 |
| Risk of bias across studies | 22 | Present results of any assessment of risk of bias across studies for the evidence base being studied. | Risk of bias; Supplementary Figure 9 |
| Results of additional analyses | 23 | Give results of additional analyses, if done (e.g., sensitivity or subgroup analyses, meta-regression analyses*, alternative network geometries studied, alternative choice of prior distributions for Bayesian analyses,* and so forth). | Subgroup and sensitivity analyses; Trial sequential analysis; Supplementary Tables 5-6 and Figures 6-7 |
|  |  |  |  |
| **DISCUSSION** |  |  |  |
| Summary of evidence | 24 | Summarize the main findings, including the strength of evidence for each main outcome; consider their relevance to key groups (e.g., healthcare providers, users, and policy-makers). | Discussion |
| Limitations | 25 | Discuss limitations at study and outcome level (e.g., risk of bias), and at review level (e.g., incomplete retrieval of identified research, reporting bias). *Comment on the validity of the assumptions, such as transitivity and consistency. Comment on any concerns regarding network geometry (e.g., avoidance of certain comparisons).* | Discussion |
| Conclusions | 26 | Provide a general interpretation of the results in the context of other evidence, and implications for future research. | Conclusion |
|  |  |  |  |
| **FUNDING** |  |  |  |
| Funding | 27 | Describe sources of funding for the systematic review and other support (e.g., supply of data); role of funders for the systematic review. This should also include information regarding whether funding has been received from manufacturers of treatments in the network and/or whether some of the authors are content experts with professional conflicts of interest that could affect use of treatments in the network. | Funding |

PICOS = population, intervention, comparators, outcomes, study design.

* Text in italics indicates wording specific to reporting of network meta-analyses that has been added to guidance from the PRISMA statement.

† Authors may wish to plan for use of appendices to present all relevant information in full detail for items in this section.

**Supplemental Table 1. Search strategy**

Up to July 25^th^, 2025

| **Search** | **Actions** | **Details** | **Query** | Results | Time |
| --- | --- | --- | --- | --- | --- |
| #1 |  |  | Search: (**β-Lactams [MeSH Terms] OR β-Lactam* [Title/Abstract] OR carbapenem* [Title/Abstract] OR meropenem [Title/Abstract] OR imipenem [Title/Abstract] OR doripenem [Title/Abstract] OR penicillin* [Title/Abstract] OR piperacillin [Title/Abstract] OR ticarcillin [Title/Abstract] OR cephalosporin* [Title/Abstract] OR cefepime [Title/Abstract] OR ceftazidime [Title/Abstract] OR cefoperazone [Title/Abstract] OR monobactam [Title/Abstract] OR aztreonam [Title/Abstract] OR Ertapenem [Title/Abstract] OR Cefazolin [Title/Abstract] OR "Clavulanic Acid*"[Title/Abstract] OR Sulbactam [Title/Abstract] OR Tazobactam [Title/Abstract])** Sort by: **Most Recent** | 233,119 | 20:51:22 |
| #2 |  |  | Search: **(Drug Administration Schedule [MeSH Terms]) OR (extended [Title/Abstract]) OR prolonged [Title/Abstract] OR continuous [Title/Abstract] OR intermittent [Title/Abstract]** Sort by: **Most Recent** | 1,439,537 | 20:52:55 |
| #3 |  |  | Search: **Administration, Intravenous [MeSH Terms] OR infusion* [Title/Abstract] OR administration* [Title/Abstract]** Sort by: **Most Recent** | 1,359,307 | 20:53:32 |
| #4 |  |  | **#2 AND #3** | 155,332 | 20:54:34 |
| **#5** |  |  | **#1 AND #4** | 4,217 | 20:54:53 |
| #6 |  |  | Search: **"randomized controlled trial" [pt] OR "controlled clinical trial" [pt] OR randomized [tiab] OR placebo [tiab] OR "drug therapy" [sh] OR randomly [tiab] OR trial [tiab] OR groups [tiab]** Sort by: **Most Recent** | 6,427,497 | 20:55:20 |
| **#7** |  |  | **#5 AND #6** | 2,516 | 20:55:39 |

**Supplemental Table 2. Characteristics of included studies**

| Characteristics of included studies | |
| --- | --- |
| Abdul-Aziz MH 2016 | |
| Risk of bias table | |
| Bias | Support for judgement |
| Random sequence generation (selection bias) | "Randomisation was performed using a computer program (http://www.randomization.com) based on blocks of four with an allocation ratio of 1:1 stratified by participating sites." |
| Allocation concealment (selection bias) | "Participants were randomly allocated to either a CI (intervention arm) or IB (control arm) treatment arm." |
| Blinding of participants and personnel (performance bias) | "Each antibiotic dose was prepared by an on-duty, unblinded ICU pharmacist in accordance with standard pharmacy practice." Patients, and study investigators were aware of treatment allocation. |
| Blinding of outcome assessment (detection bias) | "Independent investigators who were blinded to treatment allocation, patient care and management assessed the endpoints of interest." |
| Incomplete outcome data (attrition bias) | All the data was reported. |
| Selective reporting (reporting bias) | All the outcomes were reported. |
| Other bias | The sample size was calculated to achieve clinical cure. |
|  |  |
| Álvarez-Moreno CA 2024 | |
| Risk of bias table | |
| Bias | Support for judgement |
| Random sequence generation (selection bias) | The method of random sequence generation was not tell. |
| Allocation concealment (selection bias) | Randomization was performed using a sealed envelope design. Envelopes containing the information of the randomization were sealed and kept in the folder of patients until the end of the study. |
| Blinding of participants and personnel (performance bias) | This study was a nonblinded single center trial, patients, and study investigators were aware of treatment allocation. |
| Blinding of outcome assessment (detection bias) | The study investigators were aware of treatment allocation, the authors did not tell who should collect and analyze the data. |
| Incomplete outcome data (attrition bias) | All the data was reported. |
| Selective reporting (reporting bias) | All the outcomes were reported. |
| Other bias | There were three equal groups in the trial. |
| Ammar MA 2018 | |
| Risk of bias table | |
| Bias | Support for judgement |
| Random sequence generation (selection bias) | The method of random sequence generation was not tell. |
| Allocation concealment (selection bias) | "Randomization was performed using a sealed envelope design. Envelopes containing the information of the randomization were sealed and kept in the folder of patients until the end of the study." |
| Blinding of participants and personnel (performance bias) | This study was a nonblinded single‑center trial, patients, and study investigators were aware of treatment allocation. |
| Blinding of outcome assessment (detection bias) | The study investigators were aware of treatment allocation, the authors did not tell who should collect and analyze the data. |
| Incomplete outcome data (attrition bias) | All the data was reported. |
| Selective reporting (reporting bias) | All the outcomes were reported. |
| Other bias | There were three equal groups in the trial. |
|  |  |
| Angus BJ 2000 | |
| Risk of bias table | |
| Bias | Support for judgement |
| Random sequence generation (selection bias) | The method of random sequence generation was not tell. |
| Allocation concealment (selection bias) | "Patients were randomised to receive ceftazidime 120 mg/kg/day in normal saline; (Fortum1, Glaxo, Greenford, UK) either by continuous infusion or intermittent bolus injection." The method of allocation concealment was not tell. |
| Blinding of participants and personnel (performance bias) | Patients, and study investigators were aware of treatment allocation. |
| Blinding of outcome assessment (detection bias) | The authors did not tell who should collect and analyze the data, or whether he was blinded to the allocation. |
| Incomplete outcome data (attrition bias) | The authors did not tell what is the outcome data in methods. |
| Selective reporting (reporting bias) | All the outcomes were reported. |
| Other bias | The primary outcome is pharmacokinetic-pharmacodynamic evaluation, is not the mortality or clinical cure rate. |
|  |  |
| Bao H 2017 | |
| Risk of bias table | |
| Bias | Support for judgement |
| Random sequence generation (selection bias) | "the allocation status was determined by opening a sequentially numbered sealed envelope." |
| Allocation concealment (selection bias) | "an unblinded research nurse or pharmacist was responsible for the preparation of the blinded medications; the allocation status was determined by opening a sequentially numbered sealed envelope." |
| Blinding of participants and personnel (performance bias) | Patients, and study investigators were aware of treatment allocation. |
| Blinding of outcome assessment (detection bias) | The authors did not tell who should collect and analyze the data, or whether he was blinded to the allocation. |
| Incomplete outcome data (attrition bias) | All the data was reported. |
| Selective reporting (reporting bias) | All the outcomes were reported. |
| Other bias | The sample size was calculated groundlessly. Sample size relatively small. |
|  |  |
| Chytra I 2012 | |
| Risk of bias table | |
| Bias | Support for judgement |
| Random sequence generation (selection bias) | The method of random sequence generation was not tell. |
| Allocation concealment (selection bias) | "randomized using sealed opaque envelopes in one-to-one proportion without stratification" |
| Blinding of participants and personnel (performance bias) | "either continuous infusion (Infusion group, n =120) or intermittent intravenous application (Bolus group, n = 120) of open-label meropenem" |
| Blinding of outcome assessment (detection bias) | The investigators who evaluated the clinical response were aware of treatment allocation. |
| Incomplete outcome data (attrition bias) | All the data was reported. |
| Selective reporting (reporting bias) | All the outcomes were reported. |
| Other bias | None. |
|  |  |
| Cotrina-Luque J 2016 | |
| Risk of bias table | |
| Bias | Support for judgement |
| Random sequence generation (selection bias) | "the list of random numbers provided by the coordinator center at the start of the study.The randomization code for each patient consisted of three letters (corresponding to the hospital) and three numbers (corresponding to the randomization sequence)." |
| Allocation concealment (selection bias) | "Eligible patients were allocated to one of the two study groups by the Pharmacy Department of each participating hospital, using the list of random numbers provided by the coordinator center at the start of the study." "Patients were randomly assigned in a 1:1 ratio to receive piperacillin–tazobactam as either continuous infusion (CI group) or standard intermittent infusion (II group, controls)." |
| Blinding of participants and personnel (performance bias) | "The solutions of active drug had the same volume, colour, and appearance as the placebo solution. The only person who knew what treatment a patient was receiving was the member of the pharmacy department designated to prepare the intravenous solutions." |
| Blinding of outcome assessment (detection bias) | "A member of the study team accurately recorded and updated the drug dispensation data in the product accounting log and the case report forms. The pharmacy investigators performed an interim analysis of random allocation, so the remaining investigators remained blinded, to promptly detect possible imbalances between the groups." |
| Incomplete outcome data (attrition bias) | All the data was reported. |
| Selective reporting (reporting bias) | All the outcomes were reported. |
| Other bias | None. |
|  |  |
| Dulhunty JM 2013 | |
| Risk of bias table | |
| Bias | Support for judgement |
| Random sequence generation (selection bias) | "opening a sequentially numbered sealed envelope." |
| Allocation concealment (selection bias) | "Randomization was stratified by institution with 1:1 allocation to treatment arm. Following study enrollment, an unblinded research nurse or pharmacist responsible for preparation of the blinded medications determined allocation status by opening a sequentially numbered sealed envelope." |
| Blinding of participants and personnel (performance bias) | "Clinical staff, data collectors, and patients were blinded to allocation status." |
| Blinding of outcome assessment (detection bias) | "data collectors, and patients were blinded to allocation status." |
| Incomplete outcome data (attrition bias) | All the data was reported. |
| Selective reporting (reporting bias) | All the outcomes were reported. |
| Other bias | The primary outcome is plasma antibiotic concentration above MIC. |
|  |  |
| Dulhunty JM 2015 | |
| Risk of bias table | |
| Bias | Support for judgement |
| Random sequence generation (selection bias) | "The QIMR Berghofer Medical Research Institute generated the random allocation sequence", but the method of random sequence generation was not tell. |
| Allocation concealment (selection bias) | "a consecutively labeled sealed opaque envelope to determine treatment allocation before study drug preparation" |
| Blinding of participants and personnel (performance bias) | "Concealment was achieved by opaque labeling and double-dummy administration with adequacy of blinding reported previously (18). Participants, treating clinicians, and study investigators undertaking study assessments or data collection were masked to treatment allocation." |
| Blinding of outcome assessment (detection bias) | "study investigators undertaking study assessments or data collection were masked to treatment allocation." "The QIMR Berghofer Medical |
|  | Research Institute managed the trial database, and conducted the data analysis according to a prespecified statistical analysis plan with independent validation" |
| Incomplete outcome data (attrition bias) | All the data was reported. |
| Selective reporting (reporting bias) | All the outcomes were reported. |
| Other bias | None. |
|  |  |
| Dulhunty JM 2024 | |
| Risk of bias table | |
| Bias | Support for judgement |
| Random sequence generation (selection bias) | Randomization was conducted using a password-protected, encrypted web-based interface with minimization and stratification by study site. The method is clearly described and appropriate. |
| Allocation concealment (selection bias) | The allocation sequence was generated by an independent statistics unit (The George Institute). Randomization was centrally controlled and concealed from investigators. |
| Blinding of participants and personnel (performance bias) | This was an open-label trial. Neither patients nor clinical staff were blinded to treatment allocation. However, the primary outcome (90-day all-cause mortality) is objective. |
| Blinding of outcome assessment (detection bias) | While the trial was not blinded, the primary outcome was death, an objective and hard endpoint. This minimizes the risk of detection bias. |
| Incomplete outcome data (attrition bias) | Only 0.7% of participants had missing primary outcome data (50/7031), and these were well-accounted for. |
| Selective reporting (reporting bias) | All prespecified primary, secondary, and tertiary outcomes were reported in accordance with the registered protocol (ClinicalTrials.gov NCT03213990). |
| Other bias | The study was well-powered (n > 7000), multicenter, and conducted across 7 countries. No other obvious sources of bias identified. |
| Fan SY 2017 | |
| Risk of bias table | |
| Bias | Support for judgement |
| Random sequence generation (selection bias) | "A computer-generated unique hospital number was assigned to each patient on admission to the hospital." |
| Allocation concealment (selection bias) | "Patients whose hospital number ended with an even number (excluding the last checking digit) were assigned to the EI group, whereas those whose hospital number ended with an odd number were assigned to the NEI group." |
| Blinding of participants and personnel (performance bias) | "The intensivists and investigators were unblinded to the allocations." |
| Blinding of outcome assessment (detection bias) | The authors did not tell who should collect and analyze the data. |
| Incomplete outcome data (attrition bias) | All the data was reported. |
| Selective reporting (reporting bias) | All the outcomes were reported. |
| Other bias | None. |
|  |  |
| Georges B 2005 | |
| Risk of bias table | |
| Bias | Support for judgement |
| Random sequence generation (selection bias) | The method of random sequence generation was not tell. |
| Allocation concealment (selection bias) | The method of allocation concealment was not mentioned. |
| Blinding of participants and personnel (performance bias) | The participants and personnel were ware of allocation of intervention. |
| Blinding of outcome assessment (detection bias) | The authors did not tell who should collect and analyze the data. |
| Incomplete outcome data (attrition bias) | All the data was reported. |
| Selective reporting (reporting bias) | All the outcomes were reported. |
| Other bias | The PK/PD parameters was the primary outcome. |
|  |  |
| Khan AB 2025 | |
| Risk of bias table | |
| Bias | Support for judgement |
| Random sequence generation (selection bias) | Computer-generated random numbers were used with stratification by antibiotic and permuted blocks of four. |
| Allocation concealment (selection bias) | Allocation was concealed using sealed opaque envelopes and carried out by research staff. |
| Blinding of participants and personnel (performance bias) | Open-label design; participants and healthcare staff were aware of treatment allocation. |
| Blinding of outcome assessment (detection bias) | Outcome assessors were not explicitly blinded; no information was provided regarding blinding of outcome assessment. |
| Incomplete outcome data (attrition bias) | All pre-specified outcomes were reported, including primary and secondary endpoints. |
| Selective reporting (reporting bias) | All enrolled patients received treatment; no attrition reported, outcomes were fully recorded. |
| Other bias | Sample size was not reached due to COVID-19; open-label and single-center design may limit generalizability. |
| Laterre PF 2015 | |
| Risk of bias table | |
| Bias | Support for judgement |
| Random sequence generation (selection bias) | This is an prospective, randomized controlled trial, but the random sequence was not mentioned. |
| Allocation concealment (selection bias) | The allocation concealment was not tell. |
| Blinding of participants and personnel (performance bias) | Patients, and study investigators were aware of treatment allocation. |
| Blinding of outcome assessment (detection bias) | Study investigators were aware of treatment allocation.The authors did not tell who should collect and analyze the data. |
| Incomplete outcome data (attrition bias) | "Overall, mortality rates were 36% (5/14), 21% (3/14) and 50% (2/4) in the three times daily, continuous infusion and CVVH groups, respectively." However, mortality rates were 36% (5/14), 14% (2/14) and 50% (2/4) in the Table 2, respectively. |
| Selective reporting (reporting bias) | All the outcomes were reported. |
| Other bias | Sample size relatively small. |
|  |  |
| Lau WK 2006 | |
| Risk of bias table | |
| Bias | Support for judgement |
| Random sequence generation (selection bias) | Random sequence was not mentioned. |
| Allocation concealment (selection bias) | "Eligible patients were randomly assigned (1:1) to receive open-label piperacillin-tazobactam by either continuous or intermittent intravenous (i.v.) infusion." Methods for allocation concealment not mentioned in the main text. |
| Blinding of participants and personnel (performance bias) | This is an open-label study. Patients, and study investigators were aware of treatment allocation. |
| Blinding of outcome assessment (detection bias) | Study investigators were aware of treatment allocation. |
| Incomplete outcome data (attrition bias) | All the data was reported. |
| Selective reporting (reporting bias) | All the outcomes were reported. |
| Other bias | None. |
|  |  |
| Lips M 2014 | |
| Risk of bias table | |
| Bias | Support for judgement |
| Random sequence generation (selection bias) | The method of random sequence generation was not mentioned. |
| Allocation concealment (selection bias) | "Patients were randomised by the random permuted blocks method to receive either a short infu-sion (bolus group) or extended infusion (extended group) regimenof imipenem/cilastatin." |
| Blinding of participants and personnel (performance bias) | This is an open-label study. Patients, and study investigators were aware of treatment allocation. |
| Blinding of outcome assessment (detection bias) | The authors did not tell who should collect and analyze the data. |
| Incomplete outcome data (attrition bias) | All the data was reported. |
| Selective reporting (reporting bias) | All the outcomes were reported. |
| Other bias | The PK/PD parameters was the primary outcome. |
|  |  |
| Maranchick NF 2024 | |
| Risk of bias table | |
| Bias | Support for judgement |
| Random sequence generation (selection bias) | Randomization was stratified according to the beta-lactam regimen. The exact method was not fully described. |
| Allocation concealment (selection bias) | No detailed description of allocation concealment was provided. |
| Blinding of participants and personnel (performance bias) | Open-label trial; patients and investigators were aware of treatment allocation. |
| Blinding of outcome assessment (detection bias) | Clinical cure assessed by physicians; blinding status of assessors not reported. |
| Incomplete outcome data (attrition bias) | Incomplete data due to early study termination and small final analysis set (18/35). |
| Selective reporting (reporting bias) | All prespecified outcomes (clinical cure, microbiological cure, resistance, etc.) were reported. |
| Other bias | Study was underpowered due to small sample size; potential confounding from co-administered antibiotics and change in therapy. |
| McNabb JJ 2001 | |
| Risk of bias table | |
| Bias | Support for judgement |
| Random sequence generation (selection bias) | This is an open-label, prospective, randomized trial, but the random sequence was not mentioned. |
| Allocation concealment (selection bias) | The allocation concealment was not tell. |
| Blinding of participants and personnel (performance bias) | This is an open-label study. Patients, and study investigators were aware of treatment allocation. |
| Blinding of outcome assessment (detection bias) | Study investigators were aware of treatment allocation.The authors did not tell who should collect and analyze the data. |
| Incomplete outcome data (attrition bias) | All the data was reported. |
| Selective reporting (reporting bias) | All the outcomes were reported. |
| Other bias | Sample size relatively small. |
|  |  |
| Mirjalili M 2023 | |
| Risk of bias table | |
| Bias | Support for judgement |
| Random sequence generation (selection bias) | "The randomization was performed based on blocks of four with an allocation ratio of 1:1 stratified by participating sites, using a computer program. The allocation schedule was generated by a statistician with no other involvement in the study." |
| Allocation concealment (selection bias) | "Participants were assigned to either intervention or control groups, using block randomization method." |
| Blinding of participants and personnel (performance bias) | "The patients, intensivists, and investigators were blinded to the allocations." |
| Blinding of outcome assessment (detection bias) | "An investigator who was blinded to treatment allocation assessed the endpoints of interest. This investigator was not working in the participating ICUs during this study." |
| Incomplete outcome data (attrition bias) | All the data was reported. |
| Selective reporting (reporting bias) | All the outcomes were reported. |
| Other bias | None. |
|  |  |
| Monti G 2023 | |
| Risk of bias table | |
| Bias | Support for judgement |
| Random sequence generation (selection bias) | "A randomization list was created with the use of computer-generated, permuted-block sequences." |
| Allocation concealment (selection bias) | "Web-based, centralized randomization was performed by the attending ICU physician" |
| Blinding of participants and personnel (performance bias) | "Patients, physicians, and study investigators were blinded to treatment allocation." |
| Blinding of outcome assessment (detection bias) | "The data collection was performed by trained personnel who did not participate in patient care and were blinded to group allocation." |
| Incomplete outcome data (attrition bias) | All the data was reported. |
| Selective reporting (reporting bias) | All the outcomes were reported. |
| Other bias | None |
|  |  |
| Naiim CM 2022 | |
| Risk of bias table | |
| Bias | Support for judgement |
| Random sequence generation (selection bias) | "Randomization of patients into two groups in a 1:1 ratio was done using a computer generated randomization list. " "The randomization sequence was generated by an independent statistician." |
| Allocation concealment (selection bias) | "patients were randomized using sequentially numbered, opaque sealed envelopes (SNOSE)15." |
| Blinding of participants and personnel (performance bias) | "Physicians and patients were aware of the treatment allocation." |
| Blinding of outcome assessment (detection bias) | "Clinical signs and symptoms of infection are documented by the attending physician on the patient's medical record." |
| Incomplete outcome data (attrition bias) | All the data was reported. |
| Selective reporting (reporting bias) | All the outcomes were reported. |
| Other bias | "The primary efficacy end-point was the mean number of days to clinical success and the percentage of patients who were clinically cured after treatment." |
|  |  |
| Rafati MR 2006 | |
| Risk of bias table | |
| Bias | Support for judgement |
| Random sequence generation (selection bias) | This is a prospective, randomised controlled clinical trial, but the random sequence was not mentioned. |
| Allocation concealment (selection bias) | The method of concealment was not revealed in the main text. |
| Blinding of participants and personnel (performance bias) | Patients, and study investigators were aware of treatment allocation. |
| Blinding of outcome assessment (detection bias) | Study investigators were aware of treatment allocation.The authors did not tell who should collect and analyze the data. |
| Incomplete outcome data (attrition bias) | All the data was reported. |
| Selective reporting (reporting bias) | All the outcomes were reported. |
| Other bias | None. |
|  |  |
| Roberts JA 2007 | |
| Risk of bias table | |
| Bias | Support for judgement |
| Random sequence generation (selection bias) | "sequence generated from a table of random numbers" |
| Allocation concealment (selection bias) | "Patients were randomized into two groups—bolus administration or continuous infusion, using sequential opaque sealed envelopes which were opened by the treating physician." |
| Blinding of participants and personnel (performance bias) | Patients, and study investigators were aware of treatment allocation. |
| Blinding of outcome assessment (detection bias) | "Clinical and bacteriological outcomes were assessed at the cessation of ceftriaxone treatment by a critical care physician blinded to the groupings and with no role in the management of the subjects." |
| Incomplete outcome data (attrition bias) | All the data was reported. |
| Selective reporting (reporting bias) | All the outcomes were reported. |
| Other bias | None. |
|  |  |
| Sakka SG 2007 | |
| Risk of bias table | |
| Bias | Support for judgement |
| Random sequence generation (selection bias) | "We randomized 20 adults into a continuous-infusion group and a short-terminfusion group." but the random sequence was not tell. |
| Allocation concealment (selection bias) | "We randomized 20 adults into a continuous-infusion group and a short-terminfusion group." but the allocation concealment was not mentioned. |
| Blinding of participants and personnel (performance bias) | Patients, and study investigators were aware of treatment allocation. |
| Blinding of outcome assessment (detection bias) | Study investigators were aware of treatment allocation.The authors did not tell who should collect and analyze the data. |
| Incomplete outcome data (attrition bias) | All the data was reported. |
| Selective reporting (reporting bias) | All the outcomes were reported. |
| Other bias | The PK/PD parameters was the primary outcome. |
|  |  |
| Zhao HY 2017 | |
| Risk of bias table | |
| Bias | Support for judgement |
| Random sequence generation (selection bias) | "Patients meeting the inclusion criteria with no presence of exclusion criteria were randomized into equally numbered groups using sealed opaque envelopes without stratification." |
| Allocation concealment (selection bias) | "Patients meeting the inclusion criteria with no presence of exclusion criteria were randomized into equally numbered groups using sealed opaque envelopes without stratification." |
| Blinding of participants and personnel (performance bias) | Patients, and study investigators were aware of treatment allocation. |
| Blinding of outcome assessment (detection bias) | Study investigators were aware of treatment allocation.The authors did not tell who should collect and analyze the data |
| Incomplete outcome data (attrition bias) | All the data was reported. |
| Selective reporting (reporting bias) | All the outcomes were reported. |
| Other bias | None. |

**Supplemental Table 3. Study design and patient characteristics of included studies**

| **Authors** | **Design** | **Double blinding** | **Antibiotics** | **Patients** | **Total pats. N.** | **CI/EI vs II N.** | **CI/EI interventions** | **II interventions** | **Primary outcome** | **Age CI/EI^*^, yr** | **Age II^*^, yr** | **Male CI/EI (N, %)** | **Male II (N. %)** | **Clinical cure definition** |
| --- | --- | --- | --- | --- | --- | --- | --- | --- | --- | --- | --- | --- | --- | --- |
| **Continuous Infusion vs Intermittent Infusion** | | | | | | | | | | | | | | |
| Álvarez-Moreno CA (2024) | Randomized 1:1 multicenter double-blinded placebo-controlled study | No | cefepime | sepsis | 25 | 12 vs 13 | administered continuously every 8 h | over 30 m intermittently every 8 h over 24 h | Mortality at discharge | 60.2±16.9 | 54.2±1.4 | 8(66.7%) | 4(30.8%) | Complete resolution of sepsis/SIRS signs; day 14 |
| Angus BJ (2000) | Randomised controlled trial | No | Ceftazidime | suspected septic aemic melioidosis | 21 | 10 vs 11 | 12 mg/kg loading dose, followed by 4 mg/kg*h | 40 mg/kg every 8 h |  | 48 (29±58) | 43 (27±73) | 4(36.4%) | 9(90%) | NR |
| Chytra I (2012) | Single-center, prospective, randomized, open-label comparative study | No | Meropenem | sepsis | 214 | 106 vs 108 | a loading dose of 2 g infused over 30 minutes followed by 4 g over 24 hours. | 2 g infused over 30 minutes every 8 hours. | clinical and microbiological efficacy | 44.9±17.8 | 47.2±16.3 | 78 (65.0%) | 83 (69.2%) | Complete/partial clinical resolution; EOT |
| Cotrina-Luque J (2016) | Randomized, double-blind parallel-group clinical trial | Yes | Piperacillin-tazobactam | suspected or proven P.aeruginosa | 78 | 40 vs 38 | an initial loading dose of 2/0.25 g for 30 min., followed by continuous infusion of 8/1 g over 24 h plus placebo given for 30 min every 8 h. | an initial loading dose of 4/0.5g followed by continuous infusion of placebo plus 4/0.5 g for 30 min every 8 h. | clinical cure or clinical improvement | 64.3±14.3 | 63.8±17.3 | 26(65%) | 20(52.6%) | Cure or improvement; EOT |
| Dulhunty JM (2013) | Prospective, multicenter, double-blind, concealed, randomized controlled trial | Yes | Piperacillin-tazobactam, meropenem, and ticarcillin-clavulanate | severe sepsis | 60 | 30 vs 30 | continuous infusion over 24 hours | intermittent bolus dosing over 30 minutes |  | 54±19 | 60±19 | 23 (76.7%) | 19 (63.3%) | Complete symptom resolution; 7-14 d after cessation |
| Dulhunty JM (2015) | Prospective, multicenter, double-blind, double-dummy, randomized controlled trial (BLING II) | Yes | Piperacillin-tazobactam, ticarcillin-clavulanate or meropenem | severe sepsis | 432 | 212 vs 220 | continuous infusion | 30-minute intermittent infusion | alive ICU-free days at day 28 | 64(54-72) | 65(53-72) | 130 (61.3%) | 135 (61.4%) | Complete symptom resolution; 14 d after cessation |
| Dulhunty JM (2024) | Open-label, randomized clinical trial (BLING III) | No | Piperacillin-tazobactam or meropenem | sepsis | 7202 | 3474 vs 3507 | continuous infusion (over 24 hours) | intermittent infusion (over 30 minutes) | all-cause mortality at 90 days | 59.3±16.4 | 59.6±16.1 | 2308 (66%) | 2300 (65%) | Course completed by day 14; no restart within 48 h |
| Georges B (2005) | Prospective, open, randomized study | No | Cefepime | nosocomial pneumonopathy | 50 | 26 vs 24 | 2 g over 12 hours, twice daily | 2 g over 30 minutes, twice daily |  | 50±17 | 46±24 | 21(80.7%) | 20(83.3%) | Complete resolution; no further antibiotics |
| Khan AB (2025) | Prospective, open-label, single-center, randomized controlled trial | No | Amoxicillin-clavulanate, Piperacillin-tazobactam, Imipenem-cilastatin, Meropenem | Critically ill patients with sepsis122 | 122 | 64 vs 58 | 24h continuous infusion (with loading dose if needed) | Intermittent bolus every 6–8h over 30 min | Clinical cure at day 14 (completion without reinitiation within 48h) | 31 (26–39) | 36 (25–50) | 41 (64%) | 34 (58.6%) | Clinical resolution by day 14; no restart within 48 h |
| Laterre PF (2015) | Prospective, two-centre, randomized, controlled study | NR | Temocillin | intra-abdominal or lower respiratory tract infections caused by Enterobacteriaceae | 28 | 14 vs 14 | loading dose (2 g) administered over 30 min followed by infusion (6 g in 48 mL at 2 mL/h) | 2 g every 8 h injected over a 30 min period |  | 68+11 | 65+15 | 11(78.6%) | 6(42.9%) | Clinical cure; definition NR |
| Lau WK (2006) | Multicenter, prospective, randomized, open-label comparative study | No | Piperacillin-tazobactam | complicated intra-abdominal infections | 258 | 128 vs 130 | a bolus of 2 g/0.250 g infused over 30 min, followed by 12 g/1.5 g over 24 h | an intermittent i.v. infusion of 3 g/0.375 g infused over 30 min every 6 h | clinical response | 50.4±16.6 | 49.3±17.8 | 81 (63.3%) | 75 (57.7%) | Cure or improvement; 10-21 d after cessation |
| Maranchick NF (2024) | prospective, randomized, open-label, randomized trial | No | Cefepime, Meropenem, Piperacillin–Tazobactam | Severe pneumonia caused by Gram-negative bacteria | 35 | 19 vs 16 | Continuous infusion (24 h) | Intermittent infusion (30 min) | Emergence of Gram-negative resistance; clinical and microbiological outcomes | 67 (49–74) | 69 (61–73) | 4 (80%) | 8 (62%) | Symptom resolution and normalized temperature/WBC; no new antibiotic within 48 h |
| McNabb JJ/ Nicolau DP (2001) | Prospective, open-label, randomized trial | No | Ceftazidime | nosocomial pneumonia | 35 | 17 vs 18 | 3 g/day was administered as a continuous infusion | 2 g 3 times/day by intermittent infusion | clinical and microbiologic outcomes | 46±16 | 56±20 | 10 (58.8%) | 13(72.2%) | Complete clinical resolution plus radiographic improvement/no progression; EOT |
| Monti G (2023) | Double-blind, randomized clinical trial (MERCY) | Yes | Meropenem | sepsis or septic shock | 607 | 303 vs 304 | a dose of 3 g over 24 hours | an equal dose divided into 3 daily boluses (ie, 1 g every 8 hours) | a composite of all-cause mortality and emergence of PDR or EDR bacteria at day 28 | 65.5±14 | 63.4±15 | 195(64%) | 209(69%) | NR |
| Rafati MR (2006) | Prospective, randomised controlled trial | No | Piperacillin | Sepsis | 40 | 20 vs 20 | 2 g over 0.5 h followed by 8 g i.v daily over 24 h | 2 g over 0.5 h followed by 3 g i.v. every 6 h over 0.5 h |  | 50.1±22.2 | 48.0±20.7 | 12(60%) | 15(75%) | NR |
| Roberts JA (2007) | Open-label, randomized controlled pilot study | No | Ceftriaxone | sepsis | 57 | 29 vs 28 | a 500 mg loading dose, and 2 g as a 24 h infusion | a 500 mg loading dose in first day, 2 g administered once a day | clinical cure and bacteriological cure | 43±19 | 52±16 | 16 (55%) | 17 (61%) | Complete symptom resolution; EOT |
| Sakka SG (2007) | Randomized, Controlled Trial | No | Imipenem-cilastatin | ICU-acquired pneumonia | 20 | 10 vs 10 | a loading dose of 1 g/1 g, followed by 2 g/2 g per 24 h for 3 days. Thereafter, intermittent doses of 1 g/1 g q8h. | 1g/1 g as a short-term infusion (infusion time, 40 min) three times daily for 3 days. |  | 62±16 | 59±16 | 5 (50%) | 6 (60%) | NR |
| Zhao HY (2017) | Single-center, prospective, randomized, comparative study | NR | Meropenem | severe sepsis or septic shock | 50 | 25 vs 25 | a loading dose of 0.5 g i.v. infused over 30 min followed by 3 g over 24 h. | the first dose of 1.5 g infused over 30 min, and then 1 g infused over 30 min for every 8 h. | clinical and microbiological results | 68.0±15.4 | 67.0±12.2 | 10 (40%) | 11(44%) | Complete/partial clinical resolution; EOT |
| **Extended Infusion vs Intermittent Infusion** | | | | | | | | | | | | | | |
| Abdul-Aziz MH (2016) | Prospective, two-centre, open labelled RCT (BLISS) | No | Cefepime, meropenem or piperacillin/tazobactam | severe sepsis | 140 | 70 vs 70 | infused over 480 minutes every 8 hours | infused over 30 minutes every 8 hours | clinical cure at 14 days | 54(42-63) | 56(41-68) | 46(66%) | 50(71%) | Complete symptom resolution; 14 d after cessation |
| Ammar MA (2018) | Prospective, randomized, nonblinded, controlled, single‑center trial | No | Meropenem | ventilator‑associated pneumonia | 60 | 30 vs 30 | 2 g diluted in 240 ml NS over 3 h/8 h | 2 g diluted in 100 ml over 30 min/8 h | the clinical outcome | 55.8±3.5 | 55.5±3.8 | 24(80%) | 23(76.7%) | Complete symptom resolution; EOT |
| Bao H (2017) | Randomized, parallel study | No | Piperacillin-tazobactam | hospital-acquired pneumonia | 50 | 25 vs 25 | 4/0.5 g administered over 3 h every 6 h | 4/0.5 g administered over 30 min every 6 h | clinical response | 69.7±5.9 | 67.0±7.8 | 14 (56%) | 15 (60%) | Cure or improvement; no additional antibiotics |
| Fan SY (2017) | Single-center, open-label, prospective study | No | Piperacillin/tazobactam | bacterial infection or neutropenic fever | 367 | 182 vs 185 | 4.5 g every 8-12 hours, and infused over 4 hours. | 4.5 g every 6-8 hour, and infused over 30 minutes | the 14-day mortality rate | 69±14.6 | 70±13.8 | 126(69.2%) | 121(65.4%) | NR |
| Lips M (2014) | Single-centre, randomised, open-label, comparative study | No | Imipenem/cilastatin | hospital-acquired pneumonia | 19 | 10 vs 9 | an loading dose of 1 g/1 g over 30 min followed by an infusion of 0.5 g/0.5 g administered over 3 h every 6 h | a dose of 1 g/1 g over 30 min every 8 h. |  | 63±21 | 57±16 | 5 (55%) | 9 (90%) | NR |
| Mirjalili M (2023) | Randomized assessor-blinded clinical trial | Yes | Ampicillin/sulbactam | sepsis and septic shock | 136 | 68 vs 68 | 9 g every 8 h by continuous (infused over 4 h) | 9 g every 8 h by intermittent (infused over 30 min) | clinical cure | 53.8±15.8 | 53.1±16.2 | 37(54.5) | 38(55.9%) | Complete resolution of infection signs, abnormal temperature, and leukocytosis; EOT |
| Naiim CM (2022) | Prospective non-blinded randomized comparative study | No | Piperacillin/tazobactam | gram-negative bacterial infections | 53 | 26 vs 27 | an extended infusion (over 4 h) every 8 h | an intermittent infusion (over 30 min) every 8 h | days to clinical success and clinical cure | 57.8±15.4 | 53.0±17.641 | 12 (46%) | 14 (52%) | Resolution/improvement with normalized temperature/WBC; EOT |

Note: EOT, end of treatment; NR, not reported; SIRS, systemic inflammatory response syndrome; WBC, white blood cell count.

**Supplemental Table 4. Grading of recommendation assessment, development and evaluation summary of findings for all outcomes**

|  | Risk-of-bias assessment | Inconsistency assessment | Indirectness assessment | Publication bias assessment | Direct certainty rating to inform the network estimate | Imprecision assessment | Final certainty rating of direct estimate | Intransitivity | Imprecision assessment | Final certainty rating of direct estimate |
| --- | --- | --- | --- | --- | --- | --- | --- | --- | --- | --- |
| All-cause Mortality | | | | | | | | | | |
| CI vs II (Direct Evidence) | Serious methodological limitations | No serious inconsistency | No serious indirectness | Publication bias undetected | Moderate | No serious imprecision | Moderate |  | | |
|  | (Rate down one level)^a^ | Do not rate down | Do not rate down | Do not rate down |  | Do not rate down |  |  |  |  |
| EI vs II (Direct Evidence) | Serious methodological limitations | No serious inconsistency | No serious indirectness | Publication bias undetected | Moderate | Serious imprecision | Low |  |  |  |
|  | (Rate down one level)^a^ | Do not rate down | Do not rate down | Do not rate down |  | (Rate down one level)^b^ |  |  |  |  |
| CI vs EI (Indirect Evidence) |  | | | | | | | Serious Intransitivity | Serious imprecision | Very Low |
|  | Low | | | | | | | (Rate down one level)^c^ | (Rate down one level)^d^ |  |

| Clinical Cure | | | | | | | | | | |
| --- | --- | --- | --- | --- | --- | --- | --- | --- | --- | --- |
| CI vs II (Direct Evidence) | Serious methodological limitations | No serious inconsistency | No serious indirectness | Publication bias undetected | Moderate | No serious imprecision | Moderate |  | | |
|  | (Rate down one level)^a^ | Do not rate down | Do not rate down | Do not rate down |  | Do not rate down |  |  |  |  |
| EI vs II (Direct Evidence) | Serious methodological limitations | No serious inconsistency | No serious indirectness | Publication bias undetected | Moderate | No serious imprecision | Moderate |  |  |  |
|  | (Rate down one level)^a^ | Do not rate down | Do not rate down | Do not rate down |  | Do not rate down |  |  |  |  |
| CI vs EI (Indirect Evidence) |  | | | | | | | Serious Intransitivity | Serious imprecision | Very Low |
|  | Moderate | | | | | | | (Rate down one level)^c^ | (Rate down one level)^e^ |  |

|  | Risk-of-bias assessment | Inconsistency assessment | Indirectness assessment | Publication bias assessment | Direct certainty rating to inform the network estimate | Imprecision assessment | Final certainty rating of direct estimate | Intransitivity | Imprecision assessment | Final certainty rating of direct estimate |
| --- | --- | --- | --- | --- | --- | --- | --- | --- | --- | --- |
| Length of ICU stay | | | | | | | | | | |
| CI vs II (Direct Evidence) | Serious methodological limitations | Serious inconsistency | No serious indirectness | Publication bias undetected | Low | Serious imprecision | Very Low |  | | |
|  | (Rate down one level)^a^ | (Rate down one level)^f^ | Do not rate down | Do not rate down |  | (Rate down one level)^h^ |  |  |  |  |
| EI vs II (Direct Evidence) | Serious methodological limitations | Serious inconsistency | No serious indirectness | Publication bias undetected | Low | Serious imprecision | Low |  |  |  |
|  | (Rate down one level)^a^ | (Rate down one level)^g^ | Do not rate down | Do not rate down |  | (Rate down one level)^i^ |  |  |  |  |
| CI vs EI (Indirect Evidence) |  | | | | | | | Serious Intransitivity | Serious imprecision | Very Low |
|  | Very Low | | | | | | | (Rate down one level)^c^ | (Rate down one level)^j^ |  |

a. The RoB assessment indicates serious methodological flaws.
b. 95% Confidence interval:(0.649,1.014). Confidence interval extends into clinically important effects in both directions.
c. The network remained open (no closed loops), so the transitivity assumption may be questionable.
d. 95% Confidence interval:(0.886,1.415). Confidence interval extends into clinically important effects in both directions.
e. 95% Confidence interval:(0.732,1.023). Confidence interval extends into clinically important effects in both directions.
f. On visual inspection, the forest plot shows large differences in point estimates on both sides of the line of no effect. I^2 = 87.0%.
g. On visual inspection, the forest plot shows large differences in point estimates on both sides of the line of no effect. I^2 = 74.3%.
h. 95% Confidence interval: −0.47 (−2.10, 1.28). Confidence interval extends into clinically important effects in both directions.
i. 95% Confidence interval: −1.32 (−4.65, 2.52). Confidence interval extends into clinically important effects in both directions.
j. 95% Confidence interval: 0.86 (−3.32, 4.54). Confidence interval extends into clinically important effects in both directions.

**Supplemental Table 5. Subgroup Interaction Effects on All-Cause Mortality in β-Lactam Infusion Strategies**

| CI vs II | Interaction effect (β, 95% CI) | P | Posterior probability of being positive |
| --- | --- | --- | --- |
| Population | -0.385 (-1.313, 0.542) | 0.415 | 20.10% |
| Antibiotic class | -0.061 (-0.376, 0.245) | 0.680 | 34.70% |
| Settings | -0.260 (-0.606, 0.114) | 0.155 | 7.60% |
| Economic statue | -0.177 (-0.649, 0.207) | 0.155 | 17.50% |
| Gram infection | 0.237 (-0.205, 0.659) | 0.849 | 86.70% |
| Masking | -0.070 (-0.443, 0.276) | 0.324 | 34.70% |
| Mortality recording time | 0.228 (-0.254, 0.714) | 0.824 | 83% |
| Scope of study | -0.149 (-0.537, 0.297) | 0.286 | 22.20% |
| EI vs II |  |  |  |
| Population | 0.488 (-0.154, 1.165) | 0.133 | 93.40% |
| Antibiotic class | -0.337 (-0.855, 0.166) | 0.186 | 9.40% |
| Settings | 0.407 (-0.104, 0.929) | 0.119 | 94.30% |
| Economic statue | -0.201 (-0.817, 0.436) | 0.275 | 24.40% |
| Gram infection | 0.014 (-1.242, 1.283) | 0.512 | 50.50% |
| Masking | 0.524 (-0.281, 1.365) | 0.902 | 90.10% |
| Mortality recording time | 0.468 (-0.389, 1.337) | 0.859 | 86.20% |
| Scope of study | -0.214 (-1.165, 0.733) | 0.328 | 32.10% |

**Supplementary Table 6.** Leave-one-out sensitivity analysis excluding the BLING III trial

| **Comparison** | **Full model RR (95% CrI)** | **Excluding BLING III RR (95% CrI)** |
| --- | --- | --- |
| Mortality: CI vs II | 0.85 (0.66–0.98) | 0.78 (0.60–0.96) |
| Clinical cure: CI vs II | 1.10 (1.03–1.20) | 1.09 (1.01–1.23) |
| Mortality: CI vs EI, indirect comparison | 1.06 (0.73–1.44) | 0.98 (0.66–1.41) |
| Clinical cure: CI vs EI, indirect comparison | 0.86 (0.70–1.05) | 0.85 (0.69–1.06) |

**Supplemental Figure 1. Network Plot**

**Supplemental Figure 2. Forest Plot of Pairwise Comparisons for *All-Cause Mortality***

**2-1 CI vs II (Random effect model)**

**2-2 EI vs II (Random effect model)**

**Supplemental Figure 3. Forest Plot of Pairwise Comparisons for *Clinical Cure***

**3-1 CI vs II (Random effect model)**

**3-2 EI vs II (Random effect model)**

**Supplemental Figure 4. Forest Plot of Pairwise Comparisons for *Length of ICU stay***

**4-1 CI vs II (Random effect model)**

**4-2 EI vs II (Random effect model)**

**Supplemental Figure 5. Forest Plot of Pairwise Meta-Analysis for Hospital Length of Stay (CI vs II)**

**Supplemental Figure 6. Forest Plot of Subgroup Analyses for Primary Clinical Outcome**

**6-1 CI vs II**

**6-2 EI vs II**

**Supplemental Figure 7. Trial sequential analysis (TSA) of all-cause mortality comparing β-lactam infusion strategies. Continuous infusion versus intermittent infusion (A) and extended infusion versus intermittent infusion (B).**

**Supplemental Figure 8. Risk of Bias Assessment**

**Supplemental Figure 9. Funnel Plot**

**Supplemental Figure 10.** Bayesian posterior distributions for the indirect comparison of continuous infusion versus extended infusion of β-lactam antibiotics on all-cause mortality

A. The cumulative posterior distribution of the estimated RR, with the y-axis corresponding to the probability that the RR is less than or equal to the value on the x-axis. The darker blue-gray area indicates the cumulative probability up to RR = 1. The dashed vertical line indicates the median. B. The full posterior distribution of the estimated RR, with the dashed vertical line indicating the median value. The tan area (RR < 1) represents the posterior probability that continuous infusion is associated with lower mortality than extended infusion, and the orange area (RR ≥ 1) represents the probability that extended infusion is superior. The dotted line at an RR of 1 indicates no treatment effect. The posterior probability that extended infusion is superior to continuous infusion in reducing all-cause mortality is 63.7%.
